# Evaluation of the effectiveness of tea tree oil in treatment of *Acanthamoeba* infection

**DOI:** 10.1007/s00436-017-5377-2

**Published:** 2017-01-26

**Authors:** Edward Hadaś, Monika Derda, Marcin Cholewiński

**Affiliations:** Department of Biology and Medical Parasitology, Poznan University of Medical Sciences, 10 Fredry Street, 61-701 Poznan, Poland

**Keywords:** Acanthamoebiasis, Experimental therapy, Tea tree oil

## Abstract

Eye diseases caused by amoebae from the genus *Acanthamoeba* are usually chronic and severe, and their treatment is prolonged and not very effective. The difficulties associated with therapy have led to attempts at finding alternative treatment methods. Particularly popular is searching for cures among drugs made of plants. However, no substances with total efficacy in treating *Acanthamoeba* keratitis have been identified.

Results of our semi in vivo studies of tea tree oil simulating eyeball infection demonstrated 100% effectiveness in the case of both trophozoites and cysts of amoebae from the genus *Acanthamoeba*. The action of tea tree oil indicates that this is the first substance with a potential ability to quickly and effectively remove the amoebae from the eye. Tea tree oil has the ability to penetrate tissues, which allows it to destroy amoebae in both the shallow and deep layers of the cornea. The present research into the use of tea tree oil in the therapy of *Acanthamoeba* infection is the first study of this type in parasitology. It offers tremendous potential for effective treatment of *Acanthamoeba* keratitis and other diseases caused by these protozoa.

## Introduction

Free-living amoebae are the object of interest of scientists including not only biologists but also medical doctors, geneticists, microbiologists, cytologists and phitopharmacologists. Such a wide group of persons researching amoebae from the genus *Acanthamoeba* is due, above all, to the potentially pathogenic properties of amoebae capable of causing very serious diseases and problems associated with their treatment. Free-living amoebae from the genus *Acanthamoeba* are the aetiological factor in many disorders. They cause *Acanthamoeba* keratitis (AK), granulomatous amoebic encephalitis (GAE) and inflammation of the lungs (AP) and other tissues (such as the skin, adrenal glands, mandible, ears).

The main and most frequent disease caused by amoebae is *Acanthamoeba* keratitis. The first cases of amoebic invasion of the cornea were described in the year 1974 (Naginton et al. 1974). In the next few years, only a small number of AK cases were described, but the morbidity quickly rose after 1981.

The estimated number of infections is currently counted in the thousands. The majority of AK cases (85–88%) are connected with wearing contact lenses (Dart et al. 2009). Among this group of patients, 88% cases are associated with wearing hydrogel lenses, and 12% with using rigid lenses. Seal (2003) reports that the annual probability of contracting AK for persons wearing contact lenses is 1:30,000. AK is usually diagnosed in the advanced stage of the disease.

So far, the most effective drugs used to combat amoebae have been chlorhexidine, dibrompropamidine, pentamidine and polyhexamethylene biguanide (PHMB). In vitro tests have shown that PHMB destroys trophozoites, while chlorhexidine destroys cysts (Hammersmith 2006). The treatment of amoebic infection of the cornea is extremely difficult and protracted (Yoder et al. 2012). Currently, nowhere in the world are there drugs with marketing authorisation which are safe and effective in treating AK. The chronic nature of the infection with *Acanthamoeba* is associated mostly with the presence of cysts (Dart et al. 2009; Kosik-Bogacka et al. 2010; Seal 2003). Most drugs show high human toxicity and cause undesirable effects. For this reason, researchers seek alternative substances which could be used to treat AK. An upswing of interest in natural plant products whose secondary metabolites may potentially show amoebicidal or amoebostatic properties were seen in recent years.

## Material and methods

The studied material is a natural oil of the tea tree *Melaleuca alternifolia* (Maiden & Betche) Cheel obtained in the process of steam distillation of fresh leaves and branches of this shrub growing in Australia.

The effect of tea tree oil was tested in vitro and in vivo on the Ac55 strain of *Acanthamoeba castellanii*—isolated from a patient with *Acanthamoeba* keratitis, genotype T4 GenBank: KP120880, invading both the lungs and the brain of laboratory animals, which is representative for pathogenic strains (Derda et al. 2015). The amoebae were bred axenically on a liquid medium containing 2% Bacto-Casitone (Difco) and 10% horse plasma. Both axenic and non-axenic cultures of the Ac55 strain have the pathogenic properties.

In in vitro tests, the tea tree oil (*M. alternifolia*) in a quantity of 0.1–1 μl ml^−1^ was added to an axenic culture of amoebae containing 5 × 120 cells per ml^−1^. The increase or decrease of the number of amoebae was counted after 1 and 24 h in a Thoma cell counting chamber. The control group was a culture of amoebae without tea tree oil.

The second method used was the in vitro method studying the effects of tea tree oil on amoebae and cysts growing on a solid medium containing non-nutritional agar (NN) covered with *Aerobacter aerogenes* bacteria.

A 1-cm disc of filtration blotting paper was placed on a 10-cm dish covered with trophozoites or cysts, with a drop (approximately 25 μl) of tea tree oil added every day. The control was a culture where instead of the oil, a drop of distilled water was applied on the blotting paper.

The effect of tea tree oil on the trophozoites and cysts was observed after 30 and 90 min and over five consecutive days, from the first application of the oil on the filter.

The viability of the cysts subjected to tea tree oil was tested by rinsing them off the agar and moving to a fresh culture medium.

An in vivo test of the effect of tea tree oil on experimental amoebic infection was also carried out. The test was conducted on 2-week old BALB/c mice. Three groups of 10 animals each were tested.

Group one comprised the infected mice which were subjected to inhalation of the oil in a concentration of 0.1 μl per 1 ml of air over 30 min. The inhalation was carried out twice, over two consecutive days, starting on the day following the infection. Group two consisted of mice given tea tree oil per os in a quantity of 25 μl/mice (tea tree oil was diluted with rapeseed oil at a ratio of 1:1) over 4 days. The oil was administered for the first time on the day following the infection.

In the in vivo tests, the controls were infected mice receiving no oil in any form. Ten days after the infection, all the mice were put down and the brain and lung tissues were placed on non-nutritional agar to evaluate the effectiveness of the infection or treatment.

## Results

Table 1 presents the reduction of the number of amoebae in the liquid culture after adding tea tree oil. It was determined that a 50% reduction in the number of amoebae takes place just 1.5 h after administering 0.1 μl ml^−1^ of the oil. Administering the oil in a 0.5-μl ml^−1^ concentration causes a total destruction of all the amoebae during that time. The value of IC_50_ calculated for tea tree oil after 90 min was 0.1 μl ml^−^1, and after 24 h, IC_50_ was 0.06 μl ml^−1^.

**Table 1:** Reduction of the number of amoebae in an in vitro culture after administering tea tree oil

Drug dose	Reduction of the number of amoebae			
	1.5 h after oil administration		24 h after oil administration	
	Average number of amoebae ± SD	Inhibition (%)	Average number of amoebae ± SD	Inhibition (%)
Control	18.75 ± 1.5	0	40.5 ± 2.87	0
0.1 μl ml^−1^	9.37 ± 0.9	50.03	8.9 ± 1.6	78.03
0.5 μl ml^−1^	0	100	0	100
5.0 μl ml^−1^	0	100	0	100

**Fig. 1:**
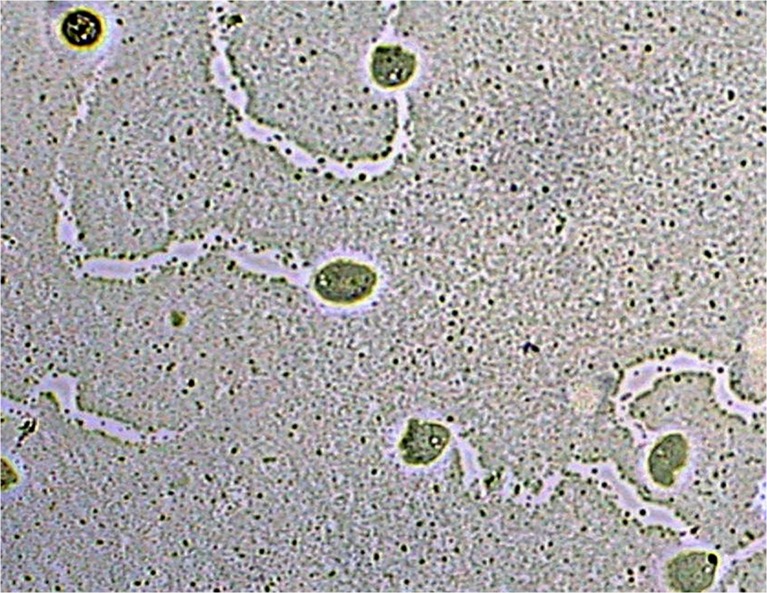
Amoebae and the traces left by their movement across the agar prior to adding the oil

**Fig. 2:**
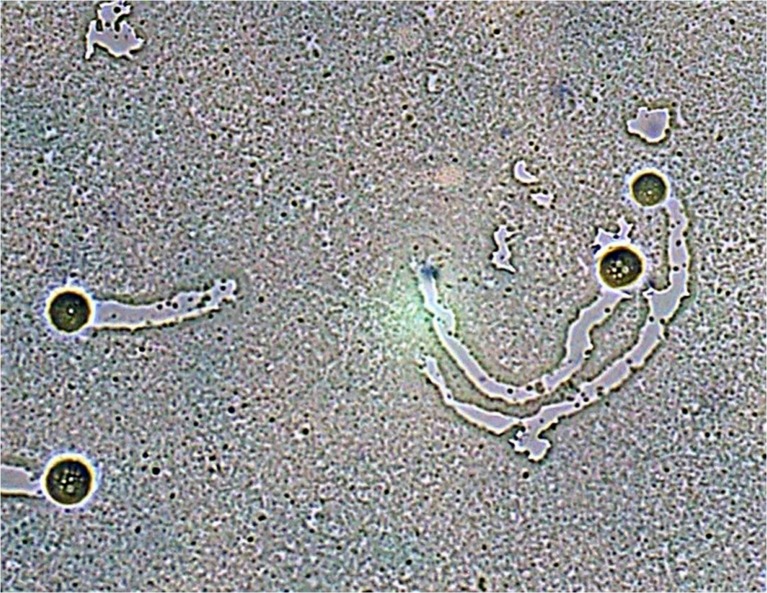
Amoebae and the traces left by their movement across the agar covered with bacteria 30 min after administration of tea tree oil

**Fig. 3:**
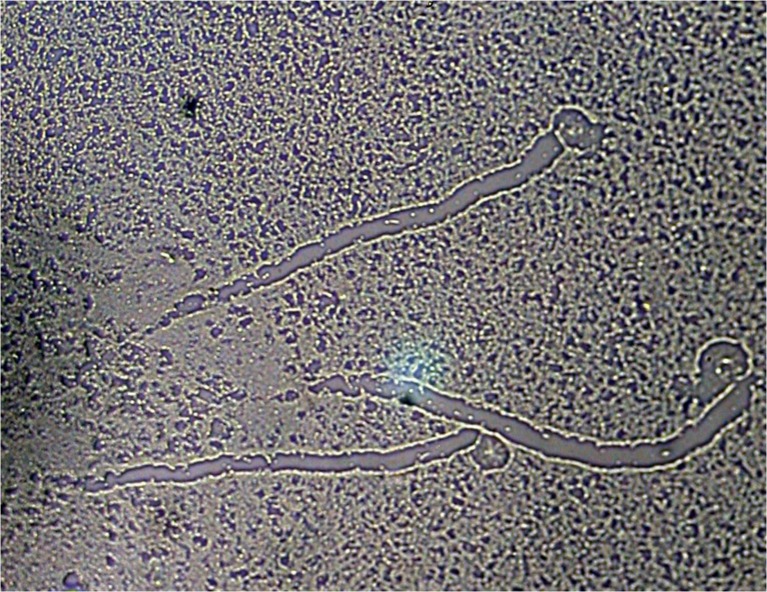
Traces left by the amoebae and their movement across the agar covered with bacteria 90 min after administration of tea tree oil

Tests of the effect of tea tree oil on trophozoites and cysts of amoebae growing on agar plates imitate conditions close to those existing in vivo in the case of *Acanthamoeba* keratitis. Researchers carrying out these tests observed rapid destruction of trophozoites after administering 25 μl of tea tree oil on a 1-cm disc of filtration blotting paper placed on agar with a culture of amoebae. Observations of the reaction of the amoebae to oil vapours are presented in Figs. 1, 2 and 3. Figure 1 shows the amoebae and the traces of their movement across the agar prior to adding the oil. Figure 2 presents the traces left by the movement and amoebae showing destructive changes 30 min after administration of the oil. Figure 3 shows the traces of the movement of the amoebae and the absence of these organisms 90 min after application of the oil.

Cysts of the amoebae subjected to a 1-day exposure to the vapours of the oil lose 70–85% of their excitation ability. After a four-day exposure, cysts completely lose the excitation ability. The effect of tea tree oil on *Acanthamoeba* cysts is presented in Table 2.

**Table 2:** Viability of *Acanthamoeba* cysts exposed to tea tree oil

Days of exposure of cysts to tea tree oil	Cyst viability (%)	Viability reduction (%)
Control from day 1–5	100	0
1 day	15–30	70–85
2 day	9–15	85–91
3 day	8–10	90–95
4 day	0–5	95–100
5 day	0	100

The results of the in vivo tests into the effect of tea tree oil in infected mice are presented in Table 3. In group one of the mice subjected to tea tree oil inhalation, the absence of the amoebae in the lungs was determined in 10 cases out of 10. The amoebae were re-isolated from the brains of these mice in four cases. In group two of the animals given the oil per os, the amoebae were re-isolated in 6 out of 10 infected mice. These organisms were mostly re-isolated from the brain tissue, and in the case of three mice, also from the lungs. The remaining animals were amoebae-free.

**Table 3:** In vivo effect of tea tree oil on infected mice depending on the method of administration

Method of tea tree oil administration	Number of animals being infected	Number of infected animals	Tissue from which the amoebae were re-isolated	
			Lungs	Brain
Inhalation	10	4	0	4
Per os	10	6	3	6
Control	10	10	10	10

All control mice were infected, and the amoebae were re-isolated from the lungs and the brain.

## Discussion

The ubiquitous presence of amoebae in the natural environment is undeniable. These cosmopolitan organisms are found in samples of soil, air and water and are isolated from animal tissues. Diagnosing an invasion of amoebae from the genus *Acanthamoeba* in the eye is difficult. Many infections of the cornea caused by viruses, fungi or bacteria give symptoms similar to those presented by an invasion by amoebae. Very often, morbid changes in the eye are misdiagnosed as infections caused by the *Herpes simplex* viruses.

*Acanthamoeba* keratitis usually affects one eye. The first symptoms of the disease are blurred vision, aversion to light and severe pain of the eye disproportionate to the damage to the cornea. There is also swelling of the conjunctiva and eyelids. The patients have red and watery eyes. Diffusive, ring-shaped or half-moon-shaped infiltrations, and less specific satellite infiltrations appear in the corneal stroma. Ring-shaped infiltrations can be few or many. The epithelium covering the corneal stroma may be intact or show punctate defects. Pus appears in the eye in the late phase of the disease. The swelling of the eye increases with the progression of the illness (Kosik-Bogacka et al. 2010).

In the case of keratitis caused by amoebae, there are no uniform treatment standards. Treating keratitis caused by amoebae is extremely difficult and long (Kaiserman et al. 2012). Besides infection, *Acanthamoeba* can transport pathogenic microorganisms into the eye as pathogenic bacteria, fungi and viruses (Hadaś et al. 2004; Scheid et al. 2008; Scheid 2014, 2015). Seal effectively completed the therapy of AK by using chlorhexidine (0.02%), propamidine (0.1%), antibiotics and administering eye drops every hour over a period of several months (Seal et al. 1995; Seal 2003). Alas, in other cases, recurrences of the disease were observed (Butler et al. 2005).

So far, various extracts and substances isolated from plants have been studied as a method of curing acanthamoebiasis (Derda and Hadaś 2015). Some of these plants are often used in natural medicine and show antiseptic and amoebicidal properties. Some arrest the development of amoebae and others cause encystation of trophozoites. Certain substances are lethal for trophozoites but are ineffective in the case of cysts. A significant and desirable trait of plant products is their amoebostatic and amoebicidal effects in the case of trophozoites and cysts. So far, the most effective plant showing both these properties has been *Artemisia annua* L. (Derda et al. 2016). However, the present study indicates that tea tree oil is an even more effective treatment in cases of infections with *Acanthamoeba*.

Tea tree oil is obtained from the plant *Melaleuca alternifolia* (Maiden & Betche) Cheel belonging to the *Myrtaceae* family (Harnischfeger and Reichling 1998). The name is derived from Captain Cook and the first colonists in Australia from 1770, who, like the Aborigines, used the leaves to brew an aromatic tea-like beverage (Foster and Tyler 1999).

Tea trees grow in Australia, mostly in the western regions, close to streams and rivers. They are branchy with white flowers. The oil can be obtained from the oil glands of tea tree leaves by using water-steam distillation.

One of the first uses of tea tree oil in medical practice was wound disinfection and treatment of wounds already infected with bacteria. Currently, tea tree oil is mostly used topically in dermatology and gynaecology. However, completely new applications have appeared, for instance as inhalations to treat the upper respiratory tract and orally in the therapy of infections of the urinary tract. Moreover, tea tree oil is used in many bacterial infections but so far has not found much use in the treatment of diseases caused by parasites. It has been used successfully in just a handful of cases, among others, to combat *Demodex* sp. in humans (Kleina Schmidt et al. 2010; Tighe et al. 2013; Cheng et al. 2015), *Bovicola ovis* in sheep (James and Callander 2012) and in vitro and in vivo against *Trypanosoma evansi* in mice (Baldissera et al. 2014).

Our semi in vivo tests of tea tree oil simulating eyeball infection have shown 100% effectiveness against *Acanthamoeba*, both trophozoites and cysts. These tests clearly show that amoebae living in the cornea can be destroyed with tea tree oil in the form of ointments, eye drops or compresses. If the effectiveness of tea tree oil is similar as in the case of semi in vivo tests on agar plates, then this would make it the first substance with a potential ability to quickly and effectively remove the amoebae from the eye without resorting to the current drastic means. Tea tree oil has the ability to penetrate tissues. This suggests that the amoebae would be destroyed in both the shallow and deep layers of the cornea. The toxicity of the oil is low (Kędzia et al. 2000a, b), and when administered in drops to the eyes of mice, it does not cause any damage.

The results of research into plants and their natural substances encourage scientists to discover new or forgotten substances with curative properties. These compounds may give beneficial effects in the treatment of less or more difficult cases. The tea tree with its oil is among such plants with a huge medicinal potential.
